# Commercially Successful Blockchain Healthcare Projects: A Scoping Review

**DOI:** 10.30953/bhty.v4.166

**Published:** 2021-04-16

**Authors:** Hao Sen Andrew Fang

**Affiliations:** 1 SingHealth Polyclinics, SingHealth, Singapore; 2 SingHealth Duke-NUS, Singapore

**Keywords:** blockchain, distributed ledger, healthcare, scoping review, success

## Abstract

**Background:**

The healthcare industry is the new frontier for blockchain technology. Given its properties of immutability and decentralization, blockchain represents an opportunity for unprecedented level of privacy and security for all stakeholders by ensuring data integrity while giving patients control over their own health data. On a backdrop of rising interest in blockchain in general and blockchain healthcare applications in particular, there has been a proliferation of blockchain healthcare projects over the past few years. The aim of this review is to identify and understand real-world blockchain healthcare projects that have attained commercial success in the highly competitive blockchain market.

**Methods and findings:**

A scoping review was performed in January 2021 on all projects in the CoinMarketCap database. Following a pre-defined inclusion and exclusion criteria, eligible projects were selected. A single reviewer then reviewed each project’s official website and whitepaper (where available) and performed data abstraction; 10 blockchain healthcare projects fulfilled the selection criteria. The review found that these projects made up 0.24% of the total number of actively tracked projects on CoinMarketCap. In terms of market capitalization, the total market capitalization for the projects was US$65,078,849, comprising less than 0.01% of the total market capitalization of all projects. Among the projects, the most frequent type was for personal health tracking.

**Conclusions:**

This review revealed that blockchain health projects currently comprise a small fraction of the overall number of commercially successful blockchain projects. However, because this sub-industry is still in its early stages, there are reasons to be optimistic that many more blockchain health projects will emerge and attain commercial success in future. Findings from this review done from an entrepreneurial perspective should help with the identification of future projects most likely to succeed.

The global blockchain technology market size was valued at US$1.5 billion in 2018 and is expected to grow at a compound annual growth rate (CAGR) of 69.4% from 2019 to 2025 (1). Over the past few years, the rising interest in blockchain has seen a proliferation of promising projects leveraging the technology. Current estimates put the number of new projects at about 200 a month (2). According to the data from CoinMarketCap (CMC), as of January 26, 2021, an estimated 8,326 blockchain projects have since been launched, with a total market capitalization nearing US$1 trillion (3).

Since the core idea of Bitcoin – the first blockchain project – was to decentralize money, many blockchain projects have focused likewise on solutions for the finance industry (4). More recently, projects have creatively started to apply blockchain across to other industries such as manufacturing, media, retail, and education (5). The healthcare industry, which is rapidly embracing digital technologies, has been touted as the next frontier for blockchain technology (6). For healthcare, blockchain technology represents an opportunity for unprecedented level of privacy and security for all stakeholders by ensuring data integrity while giving patients control over their personal health data. It comes as no surprise then that the blockchain healthcare market is forecasted to grow at an even higher CAGR of 72.0% from 2020 to 2027 (7).

Reviews of blockchain applications in healthcare have been conducted to better understand developments in the blockchain healthcare domain. Katuwal et al. and Drosatos et al. both reviewed the major use cases of blockchain in healthcare and found that most projects were limited as conceptual ideas, while a similar review by Agbo et al. also showed that there was a lack of real-world implementations (8–10). As these reviews have mostly been through the research lens, they tend to focus on projects published in research journals and conferences. This inherently excludes other projects that have not been shared in research mediums due to commercial reasons for example. Furthermore, because the analysis of projects described in these reviews included the entire spectrum from conceptual ideas to real-world implementations, the analyses of projects that actually delivered value were diluted by those that potentially could.

In this review, we aim to identify and understand real-world blockchain healthcare projects that have been commercially successful in the highly competitive blockchain market. By scoping the selection to apex projects in the blockchain field, this review should provide entrepreneurs and funders with a better understanding to judge the likelihood of success, novelty, and potential of future blockchain healthcare projects. To the best of our knowledge, this is the first formal review of blockchain healthcare applications performed through an entrepreneurial lens.

## Methods

### Study design

A scoping review was performed as the goal was to provide an overview of the successful blockchain healthcare projects instead of answering focused questions or to judge the quality of various projects. A commercially successful blockchain project was defined as a project which had attained liquidity in public financial markets.

The presented review method was carried out by defining the following activities:

1. Research questions
2. Search strategy
3. Project selection
4. Data abstraction

1. Research questions
  For this review, there were two broad questions we aimed to address:
  - Among commercially successful blockchain projects, how do the healthcare-related ones fare?
  - What are the examples of commercially successful blockchain healthcare projects?
2. Search strategy
  A list of successful blockchain projects were obtained from the CMC database. The CMC developer application programming interface (API) was used to obtain the list of cryptocurrency listings. CMC is the world’s most-referenced price-tracking website for cryptoassets in the rapidly growing cryptocurrency space. According to its website, its mission is to make cryptocurrency projects discoverable and efficient globally by providing users with unbiased, high-quality, and accurate information for drawing their own informed conclusions. Data provided from its platform have been cited by several major media outlets such as *Forbes*, *Reuters*, and *The Wall Street Journal* (11–13).
3. Project selection
  The following inclusion criteria were applied to select the suitable commercially successful healthcare projects: 1) Should be actively tracked by CMC, 2) should have a market capitalization of more than US$0, and 3) should be primarily for use in healthcare industry. Market capitalization was derived from the multiplication of the last traded token price and the number of circulating tokens. The market capitalization criteria were used to identify projects that had attracted financial investment, and those with a positive value were deemed commercially successful. Projects which did not have a website and those which were listed for less than 2 years were excluded. The 2-year mark was selected as a minimum duration of existence as this is typically the time when most projects face cash concerns, and it has been found that one-third of the new businesses do not make it past the first 2 years (14, 15).
  The project selection was performed in a stepwise manner. First, the API query was configured to extract only projects that were actively tracked in the CMC database. Next, the exported JavaScript Object Notation (JSON) list was converted to comma separated values (CSV) format using Python. Microsoft Excel was used to filter projects in the CSV file with market capitalization values of more than US$0. Finally, a single reviewer manually reviewed each project in the filtered list to identify suitable healthcare projects.
4. Data abstraction
  For data abstraction, a standardized data collection form was developed using Microsoft Excel. A review of each project’s official website and whitepaper (where available) was performed by a single reviewer knowledgeable in blockchain and the healthcare industry. The World Health Organization’s ‘Classification of digital health interventions’ was used to classify the type of healthcare project (16).

In total, 10 data elements were extracted for each project. Table 1 provides the complete list of data elements and a description of each element.

**Table 1:** List of data elements extracted and their description.

No.	Data element	Description
1	Project name	Name of the blockchain project
2	Project token symbol	Symbol used to represent token on CoinMarketCap (CMC)
3	Project website	Official website URL of the project
4	Project rank	Rank of project on CMC based on market capitalization
5	Year started	Year that the project was started
6	Market capitalization	Market capitalization on CMC
7	Market pairs	Number of markets that the project’s token was traded on
8	Project mission	Overarching mission of the project
9	Type of healthcare project	Area of healthcare industry the project is mainly applied to1
10	Blockchain platform	Blockchain platform used by the project’s solution

1 Based on a Classification of Digital Health Interventions by the World Health Organization.

## Results

### Overview

**Fig. 1:**
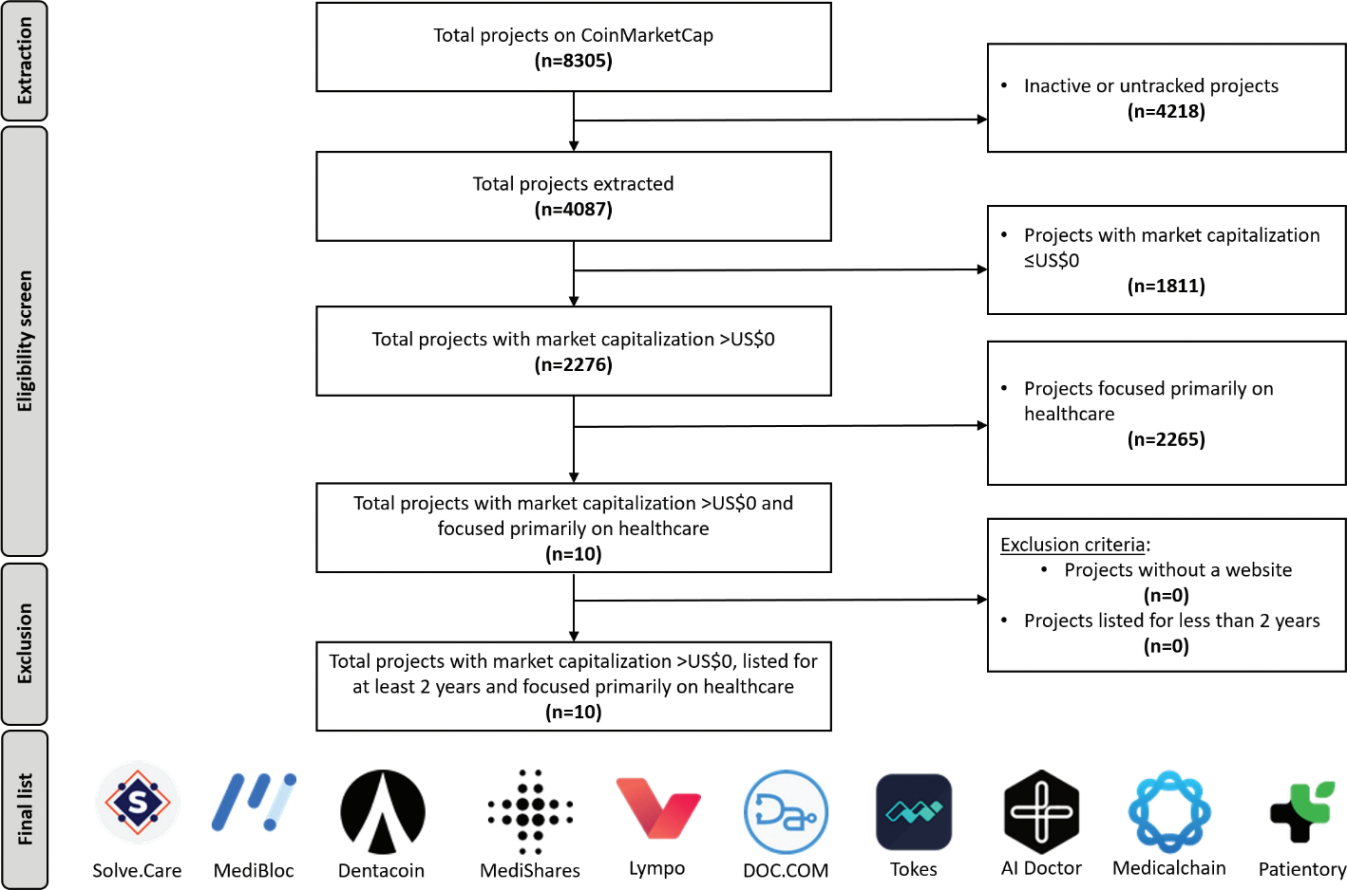
Flowchart to illustrate the screening of projects and the final list of projects for review.

As of January 23, 2021, there were a total of 8,305 blockchain projects in the CMC database. The results returned from the API query on the same date returned 4,087 actively tracked blockchain projects. From the project selection process, 10 projects were identified as successful blockchain healthcare projects for review. Figure 1 illustrates the selection process and the final list of selected projects for review. A summary of the successful blockchain healthcare projects and the data abstracted are presented in Multimedia Appendix 1.

In terms of the number of projects, healthcare projects made up a fraction (0.24%) of the total number of actively tracked projects on CMC. There were no healthcare projects among the top-100 ranked cryptocurrency projects, while there were one and five in the top-500 and top-1,000, respectively. In terms of market capitalization, the total market capitalization for healthcare projects was US$65,078,849. This made up an even smaller fraction (<0.01%) of the total market capitalization of all projects.

**Fig. 2:**
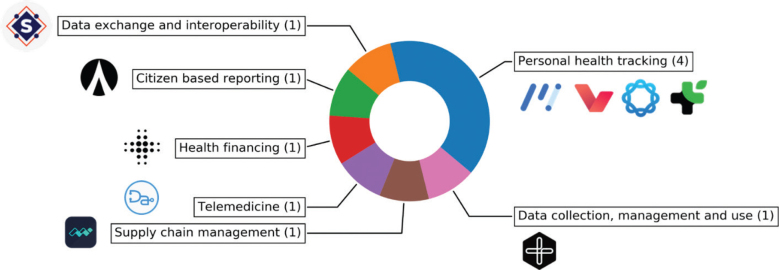
Distribution of the types of healthcare projects among those reviewed.

Among the 10 blockchain healthcare projects, the most frequent type was ‘Personal health tracking’ (*n* = 4), while the others varied from health financing to supply chain management. The projects that fell under ‘Personal health tracking’ all featured a personal health records system. Figure 2 shows the distribution of the various types of healthcare projects.

### Description of successful blockchain healthcare projects

The subsequent section provides a brief description of each of the 10 reviewed projects.

1. Solve.Care (17)
  Solve.Care is a healthcare information technology company that was founded in 2017. It is building a platform designed to use blockchain technology for coordinating care, benefits, and payments between all healthcare stakeholders. This includes patients, doctors, pharmacies, laboratories, employers, and insurers. Some use cases include appointment scheduling, specialist referrals, and insurance claims submissions. Its blockchain solution adopts a dual token mechanism – the SOLVE token and Care.Coin – with each token having distinct functionality. The SOLVE token is the native utility token required to participate in and transact on the platform. It can be utilized to pay for network fees, establish user accounts (Care.Wallets), and purchase apps (Care.Cards) from a proprietary marketplace (Care.Marketplace). The SOLVE token supply is fixed and its price is variable, as determined by market supply and demand. SOLVE tokens generate Care.Coins, a stable payment currency, which can be used for the payment of services.
2. MediBloc (18)
  MediBloc was founded by a pair of Korean entrepreneurs in 2017. It is developing a decentralized healthcare information ecosystem built on blockchain technology for patients, healthcare providers, and researchers. It allows patients to track and record all of their healthcare-related details, such as doctor visits and health records, on its blockchain platform. Apart from aggregating data, it also assigns ownership of the data to patients. This allows patients to decide which MediBloc partners they wish to share their information with. By sharing information, patients will be rewarded with the native currency MED tokens which can be used to pay for MediBloc partner products and services such as pharmaceutical purchases and insurance premiums.
3. Dentacoin (19)
  Dentacoin was started by a group of like-minded dentistry and digital transformation experts eager to reshape the dental industry by adopting blockchain technology. The aim of Dentacoin is for dental patients to write public reviews of the dentists they visit. The team has built a Trusted Reviews Platform, which allows patients to record their reviews on the blockchain, free of censorship. To ensure authenticity, it plans to implement a verified reviews feature to indicate that the review was written by people who were authorized by their dentist to write a review before their treatment began. The platform will reward patients in the form of Dentacoins tokens (DCN) for participating. The program will ultimately help users save money by using DCN to pay for their dental treatment or to purchase dental products. In the long run, it envisions that dentists could potentially consider DCN as a financial investment. For example, they could use it to remunerate employees or to pay suppliers with no middlemen and no high international transaction costs. This direct connection between producers and dentists will ultimately allow dentists to provide lower prices to patients.
4. MediShares (20)
  MediShares was created by the founders of Zhongtopia, the largest mutual aid platform in China with over 10 million users. MediShares aims to combine the traditional mutual insurance model with blockchain and smart contract, which provides a low operation cost and guarantee of compensation for risks. It is developing a blockchain platform that will serve as a decentralized marketplace for insurance. With this, participants can generate insurance smart contract, thus making it possible for anyone to find insurance coverage. On the platform, insurers are expected to profit in the form of MediShares tokens (MDS).
5. Lympo (21)
  Lympo is an Estonian blockchain startup with a mission to make the world healthier by incentivizing people to adopt healthier lifestyles. It is developing a blockchain and gamification platform which rewards participants with LYM tokens for completing certain activities such as simple challenges. For example, via its app, users are required to join six to eight daily walking and running challenges. Once a challenge is accomplished, the user will receive a reward of LYM tokens in the in-app wallet. These LYM tokens have real value and can be used to purchase quality sporting goods from an online shop – the Lympo Shop.
6. Doc.com (22)
  Doc.com was founded in 2012 in Latin America, and it provides telemedicine services to patients throughout the world. In 2018, it launched its blockchain platform. Essentially, Doc.com’s blockchain-based platform seeks to generate value from the data that patients and healthcare providers share by charging those who want to access that data. As reward to patients and healthcare providers for their contribution, they receive payment in the form of Medical Token Currency (MTC) tokens. Patients and healthcare providers can then use MTC in order to pay for services and for access to large volumes of encrypted data and valuable aggregate-level healthcare statistics.
7. Tokes (23)
  As part of the Multichain Ventures ecosystem that uses the Tokes token (TKS), the Tokes platform was founded to solve the cannabis industry’s banking problem via cryptocurrency payments. In addition to providing a payments gateway, the Tokes Platform is also building out a blockchain-based ‘track and trace’ platform for supply chain management with the capability to integrate with conventional enterprise software via API. This holistic view of the entire supply chain ensures that goods can be tracked from seed to sale with no loss or fraudulent manipulation of data along the way while complying with global supply chain management standards.
8. AI Doctor (24)
  AI Doctor was conceived as a decentralized artificial intelligence (AI) virtual doctor in 2016. The platform aims to leverage AI to analyze patients’ data to provide real-time, personalized health advice. To further incentivize users to contribute their health data, they are rewarded with AIDOC tokens on the blockchain platform. The AIDOC tokens can in turn be used to enjoy health insurance at preferential rates. These data on the platform can also be used by various organizations such as medical institutions, pharmaceuticals, and AI companies for clinical research, drug development, and AI research, respectively.
9. Medicalchain (25)
  Founded in 2017, Medicalchain aims to use blockchain technology to securely store health records and maintain a single version of the truth. Its blockchain platform will enable users to give conditional data access to different stakeholders such as doctors, hospitals, laboratories, pharmacists, and health insurers. The team is also developing a health data marketplace to allow users to negotiate commercial terms with third parties for alternative uses or applications of their personal health data. For example, putting forward their data to be used in medical research, its platform is powered by the MTN token that can be used to pay for various future services on the Medicalchain platform, such as telemedicine consultations.
10. Patientory (26)
  Founded in 2015, and based in Atlanta, Georgia, Patientory was one of the earliest healthcare projects in the blockchain space. Its key objective is to empower people to take charge of their own health. With a HIPAA-compliant platform, it enables patients, clinicians, and healthcare organizations to securely access and transfer sensitive health information while providing actionable insights to improve health outcomes. On its platform, its cryptocurrency token, PTOY, is a utility token to rent space to store healthcare data. It ensures data quality, helps to regulate payment transactions, and ensures access to the Patientory platform.

## Discussion

### Principal findings

In this first ever review of blockchain healthcare projects focusing on those that have attained commercial success, we found that healthcare projects make up a small fraction of the overall number of projects that have managed to penetrate the blockchain market, both in terms of market capitalization and number of projects. This may also be due to the fact that majority of projects continue to follow Bitcoin’s lead and target the finance industry. Also, ‘DeFi’ – a portmanteau of ‘decentralized finance’ – has become the new marketing buzzword in blockchain investor circles. As a result, these types of DeFi projects have recently attracted disproportionately large investor interest. On the contrary, the healthcare industry tends to lag in terms of adopting digital innovations, usually due to stricter regulations on new products and innovations. However on that point, it is worth noting that US national health spending is accelerating and is projected to account for almost 20% of US gross domestic product by 2028 (27). This trend will be similar for other countries. So, if governments decide to push for blockchain in healthcare, like how electronic health records were encouraged as part of the US Health Information Technology for Economic and Clinical Health (HITECH) Act, we may see healthcare easily leapfrog other industries in the adoption of blockchain (28).

The use cases of the successful blockchain healthcare projects reviewed are diverse, ranging from patient empowerment to creating mutual aid marketplaces (20, 21). With data privacy and ownership concerns becoming more prominent, it was not surprising that personal health tracking was the most frequent type of blockchain healthcare project (18, 21, 25, 26). This is a natural fit for blockchain which aims to decentralize data ownership by putting it in the hands of the end-users, instead of centralized within health institution’s databases (29). Findings from the aforementioned reviews by Katuwal et al., Drosatos et al. and Agbo et al. had also found that health data management was the most frequent use case among projects described in journals and conference publications (8–10). This indicates a healthy pipeline of projects experimenting in the area, and this may augur more innovations for the use case. This review also came across interesting projects that combined blockchain technology with other new technologies such as AI and telemedicine (22, 24). Another new technology that is being studied in combination with blockchain is Internet-of-Things (IoT). Outside this review, we researchers are also working on building infrastructure for data sharing on blockchain which can potentially integrate with IoT devices in the future (30). This appropriate blending of technologies may open up new use cases and will help blockchain projects to stand out and attract greater investor interest.

Ethereum was the most frequently used blockchain platform among the blockchain health projects. While Ethereum itself is upgrading to a new version (version 2.0) to improve scalability, other blockchain platforms are also rapidly building out new capabilities (31). For example, NEO will be adding new capabilities such as native oracles, identity management, and decentralized file storage systems to its blockchain (32). These blockchain platform enhancements will likely see more exciting new blockchain projects in future, especially healthcare-related ones that target patient data management. Separately, projects are also beginning to integrate multiple blockchains. Patientory, which was initially built on Ethereum, later integrated another blockchain platform, Dash, for its payments function (33). Medicalchain was developed on a dual blockchain structure, using both Ethereum and Hyperledger Fabric (25). In future, we may see more of such projects adopting multiple blockchains. However, more interestingly, we may expect projects to develop cross-chain interoperability where tokens can seamlessly flow from one chain to another using the same interface (34).

### Limitations

It is acknowledged that this review has its limitations. First, by only including projects that were linked to cryptocurrencies, it excludes other blockchain healthcare projects that did not use cryptocurrencies such as Estonia’s electronic health records based on blockchain technology (35). However, the advantage of this narrower scope allowed both a qualitative and quantitative comparison (based on number of projects and market capitalization) of the various healthcare projects within and also against non-healthcare projects. Second, it only searched projects from a single database – CMC. This limitation is mitigated by the fact that CMC is the leading tracking service for cryptocurrency projects, and alternative databases such as CoinGecko and ICOBench were comparatively smaller, with 6,185 and 5,728 projects, respectively, at the time of search. Furthermore, CMC was transparent with its listings criteria published on its website (36). Third, we recognize that there are other definitions of commercial success, such as revenue generated and monthly average users, apart from purely market capitalization. However, these other metrics tend to be less well reported in the public domain and therefore were not included in this review.

### Future work

This review focused on the successes. It leaves unanswered the proportion of total investment amount from healthcare industry and number of projects this small group of blockchain health projects comprise. To answer this, a subsequent review may look to include unsuccessful blockchain health projects in the analysis. Also, the sub-industry is likely at its first inning of an exciting journey of blockchain adoption in healthcare. It would therefore also be informative to continue trending its developments over the coming years.

### Conclusion

This scoping review of the commercially successful blockchain healthcare projects has revealed that such projects make up a small fraction of the overall number of projects that have penetrated the blockchain market. However, we found diverse ideas and use cases among the projects. On the backdrop of rising interest and innovation in this space, there is optimism that many more blockchain health projects will emerge and attain commercial success. Individuals with enthusiasm and knowledge in blockchain and healthcare will play an increasingly important role in helping to separate the wheat from the chaff. As the first review done from the entrepreneurial perspective, we expect this review to be a useful reference for those who take up the role and serve as a basis for future reviews to track the progress of this space.

## Supporting information

Multimedia Appendix 1. Summary of successful blockchain healthcare project and the data abstracted.
